# The extended consequences of genetic conductivity: Mating distance affects community phenotypes in Norway spruce

**DOI:** 10.1002/ece3.4616

**Published:** 2018-10-31

**Authors:** Erik Petter Axelsson, John Keith Senior

**Affiliations:** ^1^ Department of Wildlife, Fish and Environmental Studies Swedish University of Agricultural Sciences Skogsmarksgränd Umeå

**Keywords:** assisted gene flow, assisted migration, climate change, community and ecosystem genetics, genetic structure, global change, inbreeding

## Abstract

Anthropogenic landscape‐level alterations such as habitat fragmentation and long distance translocation of genetic material are currently altering the genetic connectivity and structure of forest tree populations globally. As the susceptibility of individual trees to dependent organisms is often genetically determined, it is possible that these genetic changes may extend beyond individuals to affect associated communities. To test this, we examined how variation in crossing distance among the progeny of 18 controlled crosses of Norway spruce (*Picea abies*) populations occurring across central Sweden affected chemical defense, and subsequently, a small community of galling *Adelges* aphids infecting planted trees at two common garden trails. Although crossing distance did not influence growth, vitality or reproduction in the studied population, it did influence the expression of one candidate defensive chemical compound, apigenin, which was found in higher concentrations within outcrossed trees. We also show that this variation in apigenin induced by crossing distance correlated with susceptibility to one member of the galling community but not the other. Furthermore, the effect of crossing distance on galling communities and the general susceptibility of Norway spruce to infection also varied with environment. Specifically, in the more benign environment, inbred trees suffered greater gall infection than outcrossed trees, which is contrary to general predictions that the effects of inbreeding should be more pronounced in harsher environments. These findings suggest that the effects of variation in crossing distance in forest trees can extend beyond the individual to influence whole communities.

## 1 INTRODUCTION

Genetic connectivity is an important factor determining the level of genetic variation contained within and among populations and can influence a range of important ecological and evolutionary processes (Hughes, Inouye, Johnson, Underwood, & Vellend, 2008; Kremer, Potts, & Delzon, 2014). Forest tree populations are currently experiencing anthropogenic alterations in connectivity that can be both positive, that is, increased connectivity when genetic material is translocated among populations, and negative when landscape scale changes such as fragmentation cause isolation, eroded genetic variation and inbreeding (Jump & Peñuelas, 2006; Xu, Tremblay, Bergeron, Paul, & Chen, 2012). As genetic variation relates to the adaptive potential of populations, the genetic connectivity of populations is of great concern in conservation genetics and climate change research (Aguilar, Quesada, Ashworth, Herrerias‐Diego, & Lobo, 2008; Leimu, Vergeer, Angeloni, & Ouborg, 2010). Furthermore, if foundation species such as forest trees suffer population decline or even extinction, this will also affect dependent communities (Brodie et al., 2014) but it is also possible that subtle genetic changes that do not necessarily result in extinction can affect dependent organisms.

There is a growing evidence to suggest that variation in mating distance can have important consequences for dependent organisms (Bello‐Bedoy & Nunez‐Farfan, 2011; Kariyat, Mauck, Moraes, Stephenson, & Mescher, 2012; Stephenson, Leyshon, Travers, Hayes, & Winsor, 2004). For example, inbred plants can attract more insect herbivores (Bello‐Bedoy & Nunez‐Farfan, 2011; Kariyat et al., 2012; Stephenson et al., 2004) and enhance insect development (Portman, Kariyat, Johnston, Stephenson, & Marden, 2015). While the mechanistic understanding of such inbreeding effects on plant–herbivore interactions remains largely unknown (Campbell, Thaler, & Kessler, 2013), it is possible that elevated homozygosity could influence the expression of genes regulating the expression of defensive compounds (Campbell et al., 2013; Maleck et al., 2000; Portman et al., 2015). Genetic correlations among traits are also common, and if inbreeding effects one trait this could also affect many others (Klápště et al., 2017), some of potential relevance for herbivores or associated communities. Furthermore, if the herbivore population is adapted to the local host population, outcrossing could disrupt evolutionary associations and, thereby, decrease herbivore abundance (Strauss and Karban 1994). These effects of host inbreeding can also differ among herbivore species (Bello‐Bedoy & Nunez‐Farfan, 2011; Hull‐Sanders & Eubanks, 2005) suggesting that variation in mating distance could have community level effects.

Genetic variation within foundation tree species can have important extended consequences for dependent communities, as demonstrated across numerous biological systems and ecological contexts (Whitham et al., 2003). Such effects are particularly pronounced in systems where variation is generated through introgression via interspecific hybridization (Dungey, Potts, Whitham, & Li, 2000; Jarvis et al., 2017; Pérez‐López, González‐Rodríguez, Oyama, & Cuevas‐Reyes, 2016; Wimp et al., 2004). For instance, Wimp et al. (2004) showed that the population genetic diversity generated by interspecific hybridization in a cottonwood hybrid (*Populus fremontii* × *P. angustifolia*) system explained 60% of the variation in arthropod diversity. Although such studies are highly biased toward genotypic/clonal variation (Hughes et al., 2008), they have been pivotal in highlighting the importance of genetic variation for communities and biodiversity (Whitham et al., 1999). However, we still lack an understanding of how anthropogenic changes that affect mating system and mating distance of forest trees might also affect dependent communities.

Norway spruce (*Picea abies* (L.) Karst.) is one of the most widespread and ecologically important native tree species in Europe, and as a wind‐pollinated, naturally outcrossing species, it represents an ideal system to assess the impact of mating distance on dependent communities. Previous studies show that the susceptibility of Norway spruce to gall infection is in part determined by the underlying genetics of the host and may also vary with environment (Axelsson, Iason, Julkunen‐Tiitto, & Whitham, 2015; Björkman, 2000). This suggests that changes to the genetic composition of Norway spruce generated through variable mating distance could have important consequences for galling communities, but these effects may too vary among sites. In this study, we use a set of 18 full‐sib families of Norway spruce trees (*Picea abies*) generated from controlled crosses between 10 mother trees and 12 father trees distributed across 19 different forest stands in the mid‐boreal zone of Sweden to address how variation in crossing distance affects associated communities. More specifically, we studied tree fitness and interactions with a small community of gall‐inducing Adelgid aphids in trees planted in two common gardens representing two different environments. Parental crossings consisted of an isolation‐by‐distance gradient which served as a metric of inbreeding, that is, full‐sibs generated from crossings between individuals within the same stand were considered more inbred, whereas crosses between more geographically separated individuals were considered more outbred (maximum geographical separation was 440 km). We hypothesized that (a) mating between geographically close parents should cause inbreeding depression in the progeny, and thus, that the expression of growth, vitality, reproduction, and chemical defense would positively correlate with increasing crossing distance. We also hypothesized (b) that the variation in chemical defense would translate to affect host susceptibility to galling communities, and consequently, (c) gall abundance would be negatively correlated with increased crossing distance. Lastly, we hypothesized that (d) the effects of crossing distance on galling communities would differ between two climatically different sites and (e) be specific to gall taxa, that is, affect the composition of these communities.

## 2 MATERIALS AND METHODS

### 2.1 Study system

**Figure 1:**
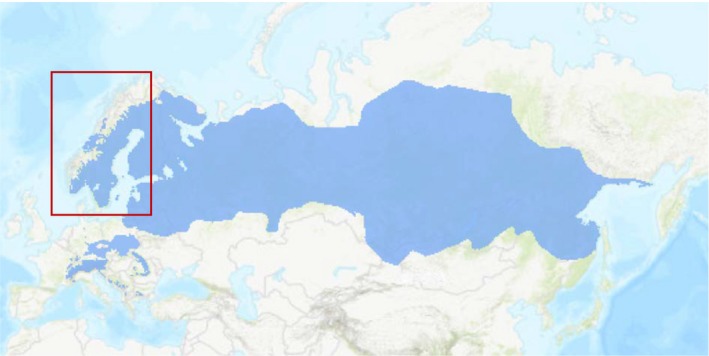
The distribution of Norway spruce (*Picea abies*) with the region of the study is highlighted in red. The distribution‐map is published with permission of EUFORGEN (http://www.euforgen.org)

Norway spruce (*Picea abies* (L.) Karst.) is one of the most widespread and ecologically important forest tree species in Europe with a distribution extending from the Atlantic ocean in Norway in the west to the Pacific ocean in Russia in the east. The natural distribution of the species is for the most part continuous, but fragmented populations occur in the southern edge of its distribution in central Europe, for example Germany, Poland, Czech Republic, Switzerland, and Austria (Figure 1). This coniferous boreal tree is predominantly wind‐pollinated, with both male and female flowers on the same individuals, but on separate organs (i.e., monoecious). With a mixed mating system, Norway spruce can produce seeds both through outcrossing (i.e., pollen dispersal as far as 4 km has been reported; Xie & Knowles, 1994) and by breeding with neighboring closely related trees, but typically avoids selfing (Burczyk, Lewandowski, & Chalupka, 2004; Finkeldey, 1995; Xie & Knowles, 1994). The genetic structure of Norway spruce shows clear latitudinal trends with decreased diversity and increased inbreeding to the north end of the distribution (Tollefsrud et al., 2009). Throughout Europe, the management of Norway spruce includes the extensive transfer of genetic material and the species has also been planted widely outside its natural range (Jansson et al., 2013).

A small community of galling adelgids from two different genera; *Sacchipantes* spp. (*S. viridis* Ratzeburg and *S. abietis* Linné) and *Adelges* spp. (*A. laricis* Vallotand *A. tardus* Dreyfus) are known to infect the fresh shoots of Norway spruce trees. The two genera represent two morphotypes. Galls of *Sacchipantes* spp., named “pineapple galls”, are characterized by a dark green color with the edges shifting toward red or brown with the infected shoot typically extending beyond the gall and eventually dying off. Galls of *Adelges* spp., named “strawberry galls”, have a yellowish color, with the infected shoot not extending beyond the gall (Hartmann, Nienhaus, & Butin, 2010). Due to these morphological differences, they can be easily distinguished in the field several years after infection and comprise a good bioindicator of adelgid community composition (Axelsson et al., 2015). The number of galls has been shown to comprise a good metric correlating with pest performance in genus *Adelges* (Björkman, 2000) and other gall‐forming arthropods (Evans, Clark, Whipple, & Whitham, 2012).

### 2.2 Experimental sites

**Figure 2:**
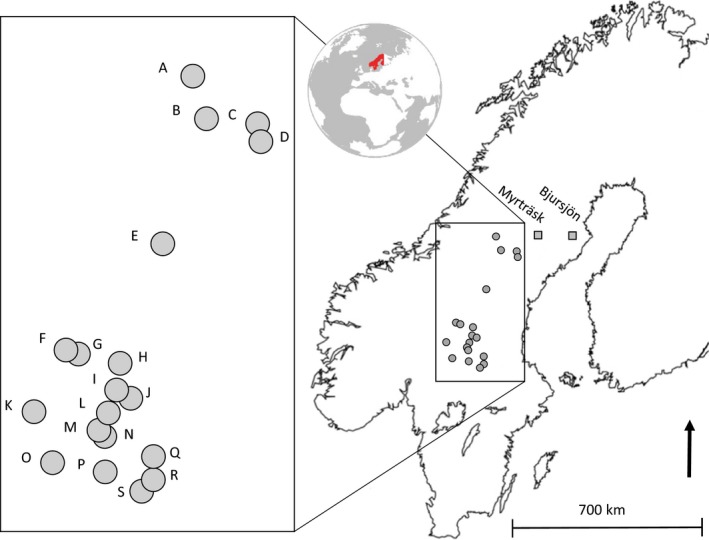
Map of Scandinavia showing the location of 19 forest stands (circles A‐S as blown up to the left) of the parental trees used in controlled crosses to generate the 18 full‐sib Norway spruce families varying in parental crossing distance and planted at two different experimental sites, one costal at Bjursjön and one inland at Myrträsk (Squares)

For this study, we build on published data showing variation in the galling communities inhibiting different Norway spruce families (Axelsson et al., 2015) by addressing if this variation could be explained by variation in isolation‐by‐distance among the parents (i.e., variation in crossing distance). To do this, we compiled geographic information of mother and father origin, and measurements of tree performance, and included data that was previously excluded (due to dead trees or because of low within family replication). This study included 176 trees from 18 different full‐sib families of Norway spruce trees planted in two common gardens, with 4–5 replicate trees of the same families planted within each garden. The full‐sib families were generated through controlled crosses (Table 1) between 10 mother and 12 father trees selected from 19 different forest stands in the mid‐boreal zone of Sweden. Parents were replicated in 1–4 crosses to generate an isolation‐by‐distance gradient which served as a metric of inbreeding, that is, full‐sibs generated from crossings between individuals within the same stand were considered more inbred, whereas crosses between more geographically separated individuals were considered more outbred (maximum geographical separation was 440 km). Trees were planted in rows in a completely randomized design in both common gardens representing different environments (Figure 2). Rows were planted 2.2 m from each other, and within rows, plants were separated by 1.7 m from each other. One site was located on the coast at Bjursjön, which is situated 40 km from the Gulf of Bothnia with relatively warm summers and mild winters (64°20'N 20°21"E, altitude 240 m) and a vegetation growing season (number of days >5°C) of 160 days. The second site was located inland at Myrträsk, which is situated 150 km from the coast with a cooler climate, harsher winters, and a shorter vegetation growing season of 140 days (64°29'N 17°54"E, altitude 490 m). Both gardens are managed by the Forest Research Institute of Sweden, and at the time of the study in August of 2013, the trees were 30 years old.

**Table 1:** The geographical location (lat, long) of 10 mother and 12 father Norway spruce trees distributed among 19 different forest stands (A‐S) used in controlled crossings to generate 18 full‐sib families varying in parental crossing distance. ID refers to the individual identification number from the Forest Research Institute of Sweden database

Family ID	Mother ♀				Father ♂				Mating distance (km)
	ID	Site	Lat	Long	ID	Site	Lat	Long	
S22H7420005	S02Y2014	C	63,80	16,45	S02S3355	O	60,52	12,78	411.7
S22H7420006	S02Z1001	E	62,62	14,58	S02S3355	O	60,52	12,78	252.8
S22H7420010	S02W3005	R	60,42	14,68	S02S3374	O	60,52	12,78	105.0
S22H7420011	S02Y2014	C	63,80	16,45	S02S3374	O	60,52	12,78	412.4
S22H7420017	S02Y2014	C	63,80	16,45	S02W2003	I	61,22	13,85	317.3
S22H7420019	S02S1001	K	60,98	12,32	S02W2003	I	61,22	13,85	86.5
S22H7420022	S02W3005	R	60,42	14,68	S02W2005	G	61,53	13,08	150.7
S22H7420029	S02Y2014	C	63,80	16,45	S02W2010	L	61,03	13,77	339.4
S22H7420046	S02W1001	F	61,58	12,90	S02W2024	H	61,47	13,92	55.6
S22H7420048	S02W2017	J	61,17	14,22	S02W2024	H	61,47	13,92	37.3
S22H7420069	S02W2029	*N*	60,87	13,68	S02W2028	M	60,87	13,67	0.6
S22H7420077	S02W4101	Q	60,62	14,67	S02W3004	P	60,48	13,80	50.3
S22H7420088	S02W3008	S	60,33	14,57	S02Y4000	D	63,68	16,55	387.4
S22H7420100	S02W3008	S	60,33	14,57	S02Z3007	A	64,28	14,93	440.0
S22H7420104	S02Z3006	A	64,28	14,93	S02Z3007	A	64,28	14,93	0.0
S22H7420110	S02Z3006	A	64,28	14,93	S02S3351	O	60,52	12,78	431.8
S22H7420123	S02Z3010	A	64,28	14,93	S02W3005	R	60,42	14,68	430.1
S22H7420108	S02Z3004	B	63,85	15,32	S02S3351	O	60,52	12,78	394.2

### 2.3 Surveys

To assess whether trees with shorter crossing distances exhibited symptoms of inbreeding depression, three parameters related to host fitness were measured for all trees across both sites: tree growth, tree vitality, and seed set (Violle et al., 2007). Tree growth was measured as bole diameter, approximately 20 cm above ground. This was preferred over the conventional Diameter at Breast Height as some trees were shorter than 130 cm. Tree vitality was estimated on a four level ordinal scale (0 = dead, 1 = severely reduced vitality, 2 = moderate reduction in vitality, 3 = healthy). Similarly, seed set was defined as the presence of cones evaluated in a four level ordinal scale (0 = none, 1 = some, 2 = substantial, 3 = abundant).

To determine whether gall abundance was related to crossing distance and whether this relationship differed between sites or gall taxa, gall abundance was surveyed on the ten lowest green branches on each tree at each site by counting the total number of galls of *Sacchiphantes* spp. and *Adelges* spp. This included both fresh galls and gall remnants from previous years and serves as a good metric for resistance, that is, gall abundance in this context reflects the plants’ ability to avoid infection but is also a good indication of performance in Adelges (Björkman, 2000) and other gall‐forming arthropods (Evans et al., 2012). The general susceptibility of Norway spruce to gall infection was expressed as the total abundance of galls irrespective of taxonomic belonging. To address a potential taxa‐specific response to mating distance, we kept the abundance of galls belonging to each genus separate, which allowed us to address community effects.

### 2.4 Chemical defense

We used previously published chemical data (Axelsson et al., 2015) on 31 different phenolic compounds collected from the site in Myrträsk to test whether inbreeding disrupts the genetic expression of major chemical defense mechanisms in Norway spruce. We specifically identified condensed tannins, apigenin, and luteolin as candidate compounds relevant for our system. Condensed tannins are of interest due to their general importance in plant defense (Juha‐Pekka, Maarit, Salminen, & Karonen, 2011), and apigenin and luteolin have previously been identified as candidate compounds of importance for the considered galling taxa (Axelsson et al., 2015). All three also occurred across families suggesting that they may be of general importance across populations of Norway spruce. See Axelsson et al. (2015) for specific details on the methods for chemical analyses.

### 2.5 Statistical analyses

All statistical analyses were conducted in JMP pro 11.2.0 (SAS Institute Inc, 2013) with all critical alpha values set to 0.05. We used generalized linear models to test whether Norway spruce traits associated with inbreeding depression (growth, vitality, reproduction, and chemical defense) and gall susceptibility (gall abundance) were influenced by among family variation in crossing distance, site, or their interaction. All models included mating distance as a covariate and site as a fixed effect, as well as their interaction. Norway spruce morphological and reproductive traits (vitality and seed set) were analyzed using an ordinal logistic distribution, while chemical defense traits were log‐transformed to meet the assumptions of a normal distribution, except luteolin which was analyzed using a Poisson distribution. Gall abundance data were also analyzed using a Poisson distribution. The chi‐squared test statistic was used to test the significance of model effects. Linear regression models were fitted to test for significant relationships between traits associated with crossing distance and gall abundance.

## 3 RESULTS

### 3.1 Effect of mating distance on trait expression

**Figure 3:**
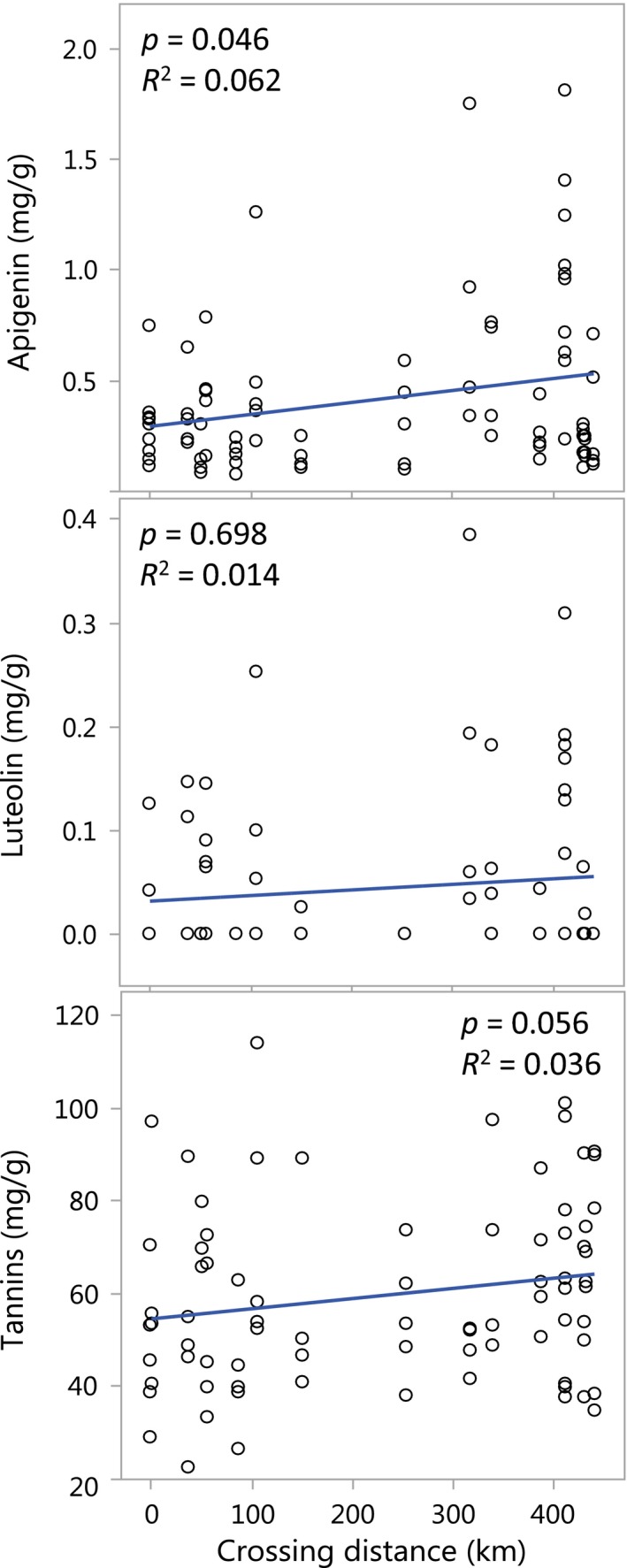
The concentration of three candidate defensive compounds in the needles of Norway spruce trees depending on parental crossing distance. *p*‐values refer to significant level of generalized linear models, and *R* ^2^‐values give the amount of variation explained by linear regression

In line with our first hypothesis, both apigenin and tannins reveal positive relations to mating distance (Figure 3) although the relation was only significant for apigenin (*p* = 0.046 and *p* = 0.056, respectively). In contrast with our first hypothesis, however, the concentration of luteolin was unaffected by crossing distance (*p* = 0.690) and, neither bole diameter, vitality, nor seed set was significantly affected by site, crossing distance, or their interaction (*p* > 0.05). Nevertheless, in line with our assumption of among site variation in climate, mean bole diameter was slightly higher in the costal site Bjursjön (99.6 mm ± 3.2) than in the inland site Myrträsk (91.6 mm ± 4.5), but the difference was not statistically significant (*p* = 0.137).

### 3.2 Chemical defense and gall infection

**Figure 4:**
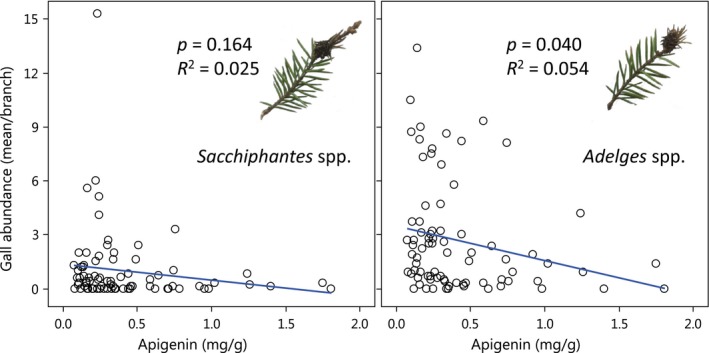
Linear regression showing the relationship between the concentration (mg/g) of apigenin in the needles of Norway spruce trees and gall abundance of *Sacchiphantes* spp. and *Adelges* spp

In support of our second hypothesis that the variation in chemical defense induced by crossing distance would translate to influence galling communities, gall abundance correlated negatively with the concentration of apigenin (Figure 4). This relation was, however, only significant for *Adelges* galls (*p* = 0.040) and not for galls of *Sacchiphantes* (*p* = 0.164).

### 3.3 Effect of site and mating distance on spruce susceptibility

**Figure 5:**
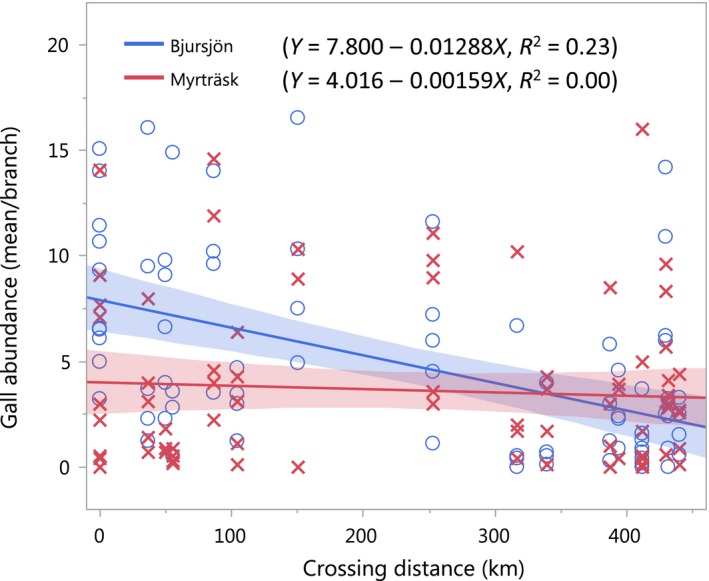
Adelgid gall abundance (mean/branch) as a measure of susceptibility of Norway spruce trees in two different sites, one costal site Bjursjön and one inland site Myrträsk, depending on parental mating distance. Each point represents one individual tree, and the shaded area around regression lines represents the model 95% confidence interval

In support of our third and fourth hypotheses, tree susceptibility to gall infection, expressed as the total abundance of galls, was significantly affected by the interaction between site and mating distance (*p* < 0.0001, Figure 5). This significant interaction was due to variable responses at the two sites, that is, increasing mating distance reduced gall abundance from about 8 to 2 galls per branch (mean 4.8 ± 0.50) in the costal site, Bjursjön, whereas gall infection was lower (mean 3.6 ± 0.43) and unaffected by mating distance in the inland site, Myrträsk (Figure 5).

### 3.4 Effect of site and mating distance on galling communities

**Figure 6:**
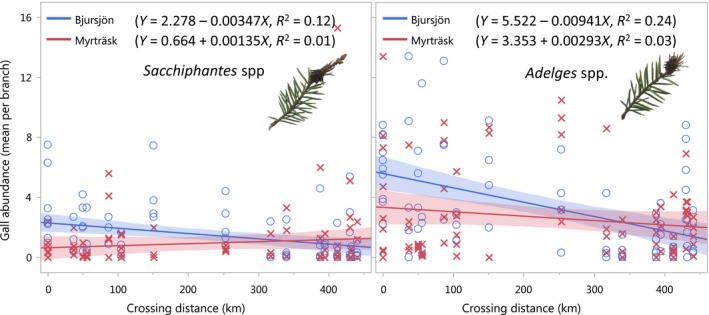
Gall abundance (mean per branch) of two different Adelges taxa (*Adelges* spp. and *Sacchiphantes* spp.) on Norway spruce trees in two different sites (one costal site Bjursjön and one inland site Myrträsk), depending on the parental crossing distance (km). Each point represents one individual tree, and the shaded area around regression lines represents model 95% confidence intervals

In support of our final hypothesis, the two galling taxa differed in their responses to site and crossing distance (Figure 6). In both cases, the interaction between site and crossing distance had a significant effect on gall abundance (*p* = 0.0009 and *p* < 0.0001, respectively), but the abundance of *Adelges* galls was predominantly influenced by crossing distance, whereas *Sacchiphantes* was more influenced by site (Table 2). *Adelges* spp. was generally more abundant on inbred compared to outcrossed trees but the significant interaction between site and crossing distance shows that this pattern was stronger in Bjursjön compared to Myrträsk

**Table 2:** Results from generalized linear models testing for the effects of site and parental crossing distance on two taxa of galling *Adelges* infecting Norway spruce trees in two different sites

	*df*	χ^2^	*p*
*Adelges* spp.			
Site	1	1.3111	0.2522
Crossing distance	1	54.3209	**<0.0001**
Site*Crossing distance	1	11.0045	**0.0009**
*Sacchiphantes* spp.			
Site	1	5.1058	**0.0238**
Crossing distance	1	1.2806	0.2578
Site*Crossing distance	1	19.4850	**<0.0001**

*p*‐values in bold indicate significant differences (*p* < 0.05).

## 4 DISCUSSION

Globally, tree populations are currently facing large‐scale genetic changes through the translocation of genetic material among populations (i.e., assisted migration) and habitat fragmentation, with uncertain consequences for dependent communities. Here, we address how variation in crossing distance in Norway spruce and environment interact to affect associated communities. In the test of our first hypothesis, we show that although crossing distance had no significant effect on growth, vitality, or reproduction in the studied Norway spruce families, it still influenced the expression of one candidate defensive chemical compound, apigenin that was higher in outcrossed trees. In line with our second hypothesis, we show that this variation in apigenin induced by crossing distance correlated with tree susceptibility to one member of the galling community but not the other. In line with our third and fourth hypotheses, we showed that crossing distance can influence the susceptibility of Norway spruce to gall infection and that this effect depends on environment. Surprisingly, the effect of crossing distance on susceptibility was only expressed in the more benign environment, which is contrary to general predictions that the effect of inbreeding should be more pronounced in harsher environments (Armbruster & Reed, 2005; Dudash, 1990; Leimu et al., 2010). Furthermore, in line with the fifth hypothesis, we show that the effect of crossing distance was specific to the gall taxa and varied with environment. This finding suggests that the effects of variation in crossing distance, that is predicted to change with ongoing landscape scale alterations (Eckert et al., 2010), can extend beyond affected populations to possibly influence entire communities.

Although naturally outcrossing species such as Norway spruce (Burczyk et al., 2004; Finkeldey, 1995; Xie & Knowles, 1994) should be sensitive to inbreeding (Husband & Schemske, 1996), we found no effect of crossing distance on morphological and reproductive traits. This may be due to the quite high genetic variation contained in the population (Androsiuk et al., 2013) which may have been sufficient to mediate some of the effects of inbreeding in the first generation progeny. Indeed, previous studies have demonstrated that inbreeding depression in Norway spruce mainly occurs in selfed offspring (Eriksson, Schelander, & Åkebrand, 1973; Skrøppa, 1996), a level of inbreeding that was not covered in this study. Furthermore, many plants abort unviable inbred offspring already as seeds and the plant material used in setting up this experiment may thus have already been purged from the most deleterious effects.

We found that crossing distance affected susceptibility so that progeny from crossings of geographically close parents suffered greater infection, at least in one of the sites. These results suggest that inbreeding in host trees plays a role in resistance to pests. Relatively few studies have examined the effects of inbreeding on nonreproductive traits, and the mechanistic understanding of inbreeding effects on defense remains largely unknown (Campbell et al., 2013). Because inbreeding results in elevated homozygosity, greater expression of recessive alleles (Jump & Peñuelas, 2006; Xu et al., 2012), and subsequent phenotypic changes, inbreeding may alter plant–insect interactions (Carr & Eubanks, 2014). For example, elevated homozygosity could influence the expression of genes involved in biochemical defense (Campbell et al., 2013; Maleck et al., 2000; Portman et al., 2015). While previous studies in this system show that among family variation in the overall composition of phenolic compounds was a poor predictor of gall infection (Axelsson et al., 2015), we demonstrate here that one of the specific compounds previously identified as a potentially important defense trait, apigenin, was indeed affected by crossing distance and could help explain variation in gall infection. It is clear from previous studies that flavonoids such as apigenin can influence feeding and the oviposition behavior of insects (reviewed in Simmonds, 2001). Another potential mechanism unexplored in this study may be variation in phenology, which has been shown to influence arthropod communities in other species (Evans et al., 2016; Hunter, 1992). Indeed, previous studies demonstrate genetic variation in the timing of bud set and bud burst in Norway spruce (Granhus, Fløistad, & Søgaard, 2009; Skrøppa, Tollefsrud, Sperisen, & Johnsen, 2010). Skrøppa et al. (2010) showed that provenances with a geographical separation similar to the separation of our parental trees were significantly different in the timing of bud set, that is, provenances originating from northern sites at latitude 64° had a bud set that was about 22 days earlier than southern provenances originating from latitude 60°.

The expression of crossing distance effects on gall communities seems to depend on environmental variation across sites. Although a climatic inference should not be pushed too far given that we are restricted to two sites, it is worth noting that the effect of crossing distance was only expressed in Bjursjön, the site with less harsh conditions, a finding that is contrary to expectations. The review by Leimu et al. (2010) suggested that the effects of inbreeding depression should become increasingly apparent under more stressful conditions, and that fitness is reduced by the synergetic effect of climate stress and eroded genetic variation (but see Armbruster & Reed, 2005). Nevertheless, given that the biotic stresses addressed here involve both plants and insect pests, which themselves may respond to changes in climate (Cudmore, Björklund, Carroll, & Lindgren, 2010), the outcome will likely depend on the response of both. In our case, the population of galling aphids seemed to benefit from a milder climate, that is, abundance was about 33% higher in the milder costal site, whereas the trees only showed a marginal and insignificant increase in growth (8.7% higher growth in the milder site). Higher abundance of galls in the milder site is in line with predictions suggesting that problems with pests and pathogens will increase in a warmer climate (Logan, Régnière, & Powell, 2003; Ramsfield, Bentz, Faccoli, Jactel, & Brockerhoff, 2016; Zhang, Lei, Ma, Kneeshaw, & Peng, 2014). If pest species expand their distribution or benefit from a milder climate while long‐lived host trees do not, a milder climate might be more stressful for the host because the pest and pathogen load might increase. Such mismatches between pest species and plants are considered a key aspect in the prediction of increased pest problems in a warming climate (Ramsfield et al., 2016; Zhang et al., 2014). Thus, an understanding of how plant genetics and climate interact to effect pest and pathogens is an important issue in genetic approaches to mediate climate change (Evans et al., 2016).

The effect of crossing distance on galling *Adelges* was specific to the different taxa and also varied with environment. This finding suggests that variation in crossing distance, which is predicted to change with ongoing landscape scale alterations (Eckert et al., 2010), can affect the composition of associated communities. The community consequences of variation in plant crossing distance are, to the best of our knowledge, not well explored. However, previous findings in herbaceous plants show that host inbreeding may have variable effects on different pests (Bello‐Bedoy & Nunez‐Farfan, 2011; Hull‐Sanders & Eubanks, 2005), suggesting the potential for community level consequences. Anthropogenic habitat modifications occur on a global scale and are currently reducing the rate of outcrossing in plant populations (Eckert et al., 2010). Studies in conservation genetics show that these changes can have detrimental effects and threaten the long term persistence of viable tree populations (Bower & Aitken, 2007; Leimu et al., 2010). We show here that the consequences of eroded genetic variation may go beyond affected populations to possibly include whole communities. Consequently, for a full understanding of the consequences of landscape scale genetic alterations, we need to consider consequences for not only affected populations but also associated communities.

The community consequences of variation in crossing distance such as seen here may also be important in a range of systems facing landscape scale changes in genetic connectivity. Regardless of whether connectivity is increased for example by assisted migration of genotypes among populations or decreased via fragmentation, there is the potential that subsequent genetic changes may influence associated communities. Throughout Europe, the management of Norway spruce includes the extensive transfer of genetic material (Jansson et al., 2013). Furthermore, assisted migration of forest trees is now considered as a management option in various regions, for example Canada (Winder, Nelson, & Beardmore, 2011), Europe (Benito‐Garzón & Fernández‐Manjarrés, 2015), and the United States (Grady et al., 2011). Although these approaches have the potential to mitigate some consequences of climate change (Aitken & Whitlock, 2013; Grady et al., 2011; Weeks et al., 2011), concerns have also been raised about the unforeseen ecological affects (Bucharova, 2017; Frascaria‐Lacoste, & Fernández‐Manjarrés, 2012). Some of these concerns are related to the maintenance of viable tree populations, as assisted migration could disrupt local adaptation to nonclimatic factors (Aitken & Whitlock, 2013; Weeks et al., 2011), such as pests and pathogens (Cudmore et al., 2010; Van der Putten, 2012), and soils (Wright, Stanton, & Scherson, 2006). However, concerns also exist regarding the maintenance of biodiversity and its effects on dependent communities (Bucharova, 2017). Here, we provide one of the first examples showing that one natural consequence of assisted migration, changes in genetic conductivity, may influence communities associated with forest trees. However, we do acknowledge the limitations of our study and argue that we need to build a better knowledge about these extended consequences (Frascaria‐Lacoste, & Fernández‐Manjarrés, 2012). We recommend that future studies should address larger communities and include both above and below ground components (Van der Putten, 2012). In future studies, there is also a need to incorporate more of the within population variation in crossing designs, and we should link ecological data to genomic validation of inbreeding and variation in genetic diversity. A merging of landscape genomics, conservation genetics, and the community and ecosystem consequences of genetic variation could help develop a better understanding of how anthropogenic landscape scale genetic changes, currently influencing forest tree populations on a global level, could influence biodiversity and eco‐evolutionary processes.

## 5 CONCLUSIONS

In this study, we demonstrate that even if mating between close relatives do not have deleterious detrimental effects on plants themselves, it can still affect communities depending on these plants for their persistence. Such effects are not commonly described in previous literature and highlight the need to better understand how genetic changes in tree populations can affect communities. Future research should, apart from establishing the community level effects of variation in mating distance, also address the driving mechanisms. Furthermore, our finding that community level consequences of variation in mating distance depend on environment suggests that these patterns are not universal and thus could change with climate change.

## CONFLICT OF INTEREST

None declared.

## AUTHORS’ CONTRIBUTION

EPA headed the conception and design, acquisition of data, and analysis and interpretation of data, and wrote the manuscript. JKS contributed substantially to analysis and interpretation of data, and revised the manuscript critically for important intellectual content.

## DATA ACCESSIBILITY

Data available from the Dryad Digital Repository: https://doi.org/10.5061/dryad.p12cn93.
